# Building a global atlas of zoonotic viruses

**DOI:** 10.2471/BLT.17.205005

**Published:** 2018-03-05

**Authors:** Dennis Carroll, Brooke Watson, Eri Togami, Peter Daszak, Jonna AK Mazet, Cara J Chrisman, Edward M Rubin, Nathan Wolfe, Carlos M Morel, George F Gao, Gian Luca Burci, Keiji Fukuda, Prasert Auewarakul, Oyewale Tomori

**Affiliations:** a Pandemic Influenza and other Emerging Threats Unit, Bureau for Global Health, United States Agency for International Development, Washington DC, United States of America (USA).; b EcoHealth Alliance, New York, USA.; c One Health Institute, School of Veterinary Medicine, University of California, Davis, California, USA.; d Metabiota, San Francisco, California, USA.; e Center for Technological Development in Health, Oswaldo Cruz Foundation, Rio de Janeiro, Brazil.; f Institute of Microbiology, Chinese Academy of Sciences, Beijing, China.; g International Law Department, Graduate Institute of International and Development Studies, Geneva, Switzerland.; h School of Public Health, Hong Kong University, Hong Kong, China.; j Department of Microbiology, Faculty of Medicine, Siriraj Hospital, Mahidol University, Bangkok, Thailand.; k Nigerian Academy of Science, University of Lagos, Lagos, Nigeria.

At the Prince Mahidol Awards Conference on 30 January 2018 in Bangkok, Thailand, policy- and decision-makers, experts, researchers, donors and private sector representatives from across the globe came together to introduce and explore the dynamics of the Global Virome Project. The project is an innovative 10-year proposed partnership to develop a global atlas of most of the planet’s naturally occurring potentially zoonotic viruses. The project aims to transform the study of emerging diseases by building an unprecedented database of viruses in their ecological contexts. This foundation of information on viral sequences, geographic ranges and host distributions will be used to drive the development of prevention efforts against future threats. This international alliance will connect the next generation of scientists, build capacity at hotspots of viral emergence and promote equitable access to data and strategies to prevent epidemics.

Despite the human and economic impact of viral epidemics, the world is not well enough prepared for the next emerging viral outbreak. Global trends indicate that new microbial threats will continue to emerge at an accelerating rate, driven by our growing population, expanded travel and trade networks, and human encroachment into wildlife habitat.^1^ Most emerging viruses are zoonotic, that is, transferred between vertebrates and humans.^2^ Nearly all zoonoses originate in mammalian or avian hosts;^3^^–^^5^ for example, type 1 human immunodeficiency virus (HIV-1) originated in chimpanzees and Ebola virus in bats.^6^ Estimations show that there are more than 1.6 million mammalian and waterfowl viruses, spanning 25 viral families known to cause human infections.^4^ Compared to just over 260 viruses known in humans,^7^ the unknown viruses represent 99.9% of potential zoonoses. These viruses usually remain undetected until they cause disease in humans.

Discovering and characterizing viruses in wildlife reservoirs is economically and technologically challenging. However, recent initiatives, including the PREDICT project of the United States Agency for International Development’s Emerging Pandemic Threats programme, have shown that systematic viral discovery, even in countries with limited laboratory infrastructure, is feasible.^8^^,^^9^ Previous studies have identified mammalian species,^10^ geographic regions^11^ and zoonotic viral transmission pathways^12^^,^^13^ with increased risk of zoonotic disease emergence. These data enable targeting of viral discovery in wildlife to expand our knowledge of likely zoonotic agents with a high potential for spillover to people. The PREDICT project has already discovered over 1000 viruses, including novel Severe Acute Respiratory Syndrome (SARS)-like coronaviruses that can infect human cells.^14^

The Global Virome Project seeks to significantly expand the scale of targeted viral discovery over a decade-long sampling and laboratory testing period.^4^ An international consortium of leading disease ecologists, public health practitioners, veterinarians, epidemiologists, biologists and laboratory scientists designed the project.^4^ The project’s working groups of ecologists, epidemiological modellers and field biologists will select sampling sites and species that harbour the greatest number of unknown zoonoses,^7^ and researchers will systematically collect and characterize viruses and their associated metadata in these areas. Protocols for the project’s implementation, including training, sampling, specimen handling, laboratory testing, reporting and data management are being developed by the PREDICT project, which has been operating for eight years in over 35 countries.^8^^,^^9^ The Global Virome Project will operate as a federation of national and regional projects led by in-country researchers, who are in turn connected to a global hub that provides standardized protocols and monitors progress. 

The Global Virome Project seeks to identify the majority of unknown viral diversity, catalogue the ecological conditions at sampling sites, and collect metadata that can be used to analyse the risk of viral spillover into humans. These data will be housed in an open-access database available to the global health community. Such a data set can be used to develop, train and test machine learning algorithms to identify patterns among viruses, classify traits that are more common in zoonoses than in other viruses, and predict which viruses have an increased risk of emerging in humans.

To ensure equitable sharing of benefits from this project, a working group for ethical, legal and social implications has been an integral part of the Global Virome Project since its inception. All research conducted as a part of the Global Virome Project will hold to ethical standards that ensure sharing, including authorship and intellectual property. Central to the ethos of the Global Virome Project is the commitment to building scientific and response capacity in the areas that need it most.

## Building capacity

Novel viruses usually emerge in regions where dense human populations and biodiversity intersect.^11^ However, these areas often have limited laboratory, surveillance and health system infrastructure, which delays detecting emerging pathogens and preventing their potential to become pandemic.^15^ Thus, to be effective, pandemic prevention should take place at the source of viral spillover events, before they spread regionally.^16^ The Global Virome Project seeks to make data on novel potential zoonotic viruses available to public health agencies that face undiagnosed illness in humans and animals. The project has the potential to benefit human and animal health through broad measures, such as: (i) using polymerase chain reaction assays for an expanded diversity of potentially zoonotic viral families to shorten the time between outbreak detection and pathogen identification; (ii)  strengthening global epidemic preparedness through investment and training in epidemiological surveillance, field biology, laboratory techniques and biosafety; (iii) identifying high-risk pathogens in wildlife populations that have high contact with people (for example hunted species and peridomestic species); (iv) establishing sample biobanks, making data and samples available for public health risk assessments and mitigation as well as more detailed pathogen studies; and (v) identifying intervention strategies for human behaviours that increase the risk of novel viral spillover.

## Enhancing our understanding

The Global Virome Project will catalyse new approaches to identify the viruses that represent the greatest threat to human or animal health. The project will use artificial intelligence across the largest viral data set ever assembled, similar to machine learning techniques that are used in genomics to identify gene function, expression and disease biomarkers.^17^ The project will use a risk assessment framework that includes data on viral phylogeny, host traits and ecological conditions where the virus exists, as well as a series of viral characteristics known to be associated with spillover, to triage viruses for further characterization.^4^ The scale of the project’s viral testing will also enable piloting and enhancing novel testing platforms technologies, such as virome capture and sequencing.^18^

The project is ambitious but feasible, enabled by technological developments that allow for rapid and affordable genetic and viral sequencing. The project is time-bound and limited in scope and has tangible progress metrics. In the past, the Human Genome Project, another ambitious science project, sequenced and mapped the human genome, starting with a focus on the genes with the greatest relevance to people. The ultimate success of the Human Genome Project is in the medical advances made after the project’s conclusion. Similarly, the Global Virome Project aims to focus testing on the minimum number of mammalian and waterfowl samples that have the greatest potential of harbouring viruses with zoonotic potential. The exclusion of invertebrates, plants, fish and other hosts from the project’s core focus is a deliberate intent to address zoonotic disease. The legacy of the Global Virome Project will probably consist of the countermeasures, diagnostics, vaccines, policies and systems that it enables. Thus, this project has the potential to achieve for pandemics and large-scale epidemics what the Human Genome Project is just beginning to do for personalized medicine.^19^

## Novel countermeasures

The development of countermeasures to viral threats requires significant time and investment, and it is unlikely that these would be developed during the initial Global Virome Project phases. The recently launched Coalition for Epidemic Preparedness Innovations represents a critical step to address known viral threats, such as the Middle East respiratory syndrome coronavirus, Lassa fever and Nipah virus, for which vaccine or countermeasure development is challenging.^20^ The Global Virome Project aims to complement the coalitions’ innovations by characterizing the size, structure and composition of the pool of unknown viruses related to the viral targets on which the coalition is focused. For example, if a candidate vaccine against Middle East respiratory syndrome could be tested against hundreds of near relatives of that same syndrome, vaccines that have broader prevention capacity could be selected and rolled out to provide better protection against future emergence of this syndrome. This approach could enhance biotechnological efforts to move from single-virus countermeasures to ones that target a whole family of viruses.^21^

## Project investment

In a single outbreak in one year, the Severe Acute Respiratory Syndrome virus wiped between 10 and 50 billion United States dollars (US$) of value from Asian stock markets due to disrupted trade and commerce.^22^^,^^23^ Influenza pandemics are estimated to cause an average of US$ 570 billion in economic damages per year to the global economy,^24^ and these costs will rise as our economies expand and become more interconnected. The Global Virome Project will cost US$ 1.2 billion, which is less than 0.2% of this estimated loss^4^ and less than the estimated US$ 2.2 billion loss in gross domestic product due to forgone economic growth in Guinea, Liberia, Sierra Leone in 2015 alone during the 2013–2016 Ebola virus disease outbreak.^25^ Much like the Global Fund, Gavi, the Vaccine Alliance and other international public health initiatives, the Global Virome Project will likely rely on a broad mix of funding streams from governments, development agencies, research agencies, private foundations and industries. Considering the increasing inevitability of pandemics and their substantial economic impact, the next generation of scientists and field workers trained through this project will have the capacity to monitor viral evolution throughout the coming years. Furthermore, the project’s open database will catalyse technological advances in risk assessment, diagnostics and countermeasures.
